# Premature Burial Precautions

**Published:** 1912-09-21

**Authors:** Jas. R. Williamson

**Affiliations:** 100 Chedington Road, Upper Edmonton, N.


*To the Editor of* The Hospital.

### Premature Burial Precautions.

*To the Editor of* The Hospital.

Sir,—The anxiety manifested by the late Mr. Thomas Henry Keesing, reported in The Hospital of last week, Mr. Nobel, the millionaire, Lord and Lady Burton, Colonel Sandys, Miss Frances Power Cobbe, Mr. Herbert Spencer, Dr. Charles Bell Taylor, the eminent oculist, and many others too numerous to mention, is due to the great uncertainty as to the ordinary signs of death relied upon by physicians. It cannot be too generally known that putrefactive decomposition is the only really trustworthy proof of death, and the late Sir Henry Thompson, F.R.C.S., said that this condition "should always be verified before a certificate of death is signed." It should also be remembered that testamentary instructions for the prevention of burial alive are often not seen until after the funeral, when the contents of the will are first made known by the family solicitor. An attempt, however, will be made in Parliament at the earliest favourable opportunity to remedy the evil complained of. The existing system of giving death certificates without personal medical verification of the fact of death, by means of scientific tests, is a very unsatisfactory guarantee against the burial of living, though apparently dead, persons. It is this amazing defect in the law that the Deaths Registration and Burials Bill aims at removing, and, not being a party question, will, it is hoped, soon be considered by Parliament and placed on the statute-book. This will, however, greatly depend upon the amount of moral and material support that is given to the Association for the Prevention of Premature Burial, the promoters of the Bill. To those readers of your admirable journal who desire to help forward this important and urgently needed reform, I should be pleased to send literature on the subject on receiving an envelope, stamped and addressed. Thanking you for your kindness.—I am, Sir, your obedient servant,

Jas. R. Williamson.

100 Chedington Road, Upper Edmonton, N.

